# Homoharringtonine combined with cladribine and aclarubicin (HCA) in acute myeloid leukemia: A new regimen of conventional drugs and its mechanism

**DOI:** 10.1155/2022/8212286

**Published:** 2022-07-13

**Authors:** Fenglin Wang, Min Xie, Pan Chen, Dan Wang, Minghua Yang

**Affiliations:** ^1^Department of Pediatrics, Third Xiangya Hospital, Central South University, Changsha 410013, Hunan, China; ^2^Hunan Clinical Research Center of Pediatric Cancer, Changsha 410013, Hunan, China; ^3^Department of Pediatrics, Xiangya Hospital, Central South University, Changsha 410008, China; ^4^Hunan Cancer Hospital and The Affiliated Cancer Hospital of Xiangya School of Medicine, Central South University, Changsha 410013, China

## Abstract

**Objective:**

The prognosis of children with refractory acute myeloid leukemia (AML) is poor. Complete remission (CR) is not always achieved with current salvage chemotherapy regimens before transplantation, and some patients have no chance of transplantation. Here, we aimed to describe a new regimen of conventional chemotherapy drugs (homoharringtonine, cladribine , and aclarubicin (HCA)) for refractory AML and its mechanism in vitro.

**Methods:**

We retrospectively collected the clinical data of 5 children with primary refractory AML using HCA as reinduction chemotherapy, and CR rates, adverse reactions, and disease-free survival (DFS) were analyzed. The effects of homoharringtonine, cladribine, and aclarubicin alone or in combination on the proliferation of HL60 and THP1 cells were analyzed by CCK-8 assay. Furthermore, CCK-8 was used to determine the effects of HCA, alone or in combination with apoptosis inhibitors, necroptosis inhibitors, ferroptosis inhibitors, or autophagy inhibitors, on the proliferation of HL60 and THP1 cells and to screen for possible HCA-mediated death pathways in AML cells. The pathway of HCA-mediated AML cell death was further verified by Hoechst/PI staining, flow cytometry, and Western blotting.

**Results:**

After 2 cycles of conventional chemotherapy, none of the 5 children with AML achieved CR and were then treated with the HCA regimen for two cycles, 4 of 5 achieved CR, and another child achieved CR with incomplete hematological recovery (CRi). After CR, 3 children underwent hematopoietic stem cell transplantation (HSCT), and only 2 of them received consolidation therapy. As of the last follow-up, all 5 patients had been in DFS for a range of 23 to 28 months. The inhibition rate of homoharringtonine, cladribine, and aclarubicin in combination on HL60 and THP1 cells was significantly greater than that of a single drug or a combination of two drugs. We found that inhibitors of apoptosis and necroptosis were able to inhibit HCA-mediated cell death but not ferroptosis or autophagy inhibitors. Compared with the control group, the number of apoptotic cells in the HCA group was significantly increased and could be reduced by an apoptosis inhibitor. Western blot results showed that PARP, caspase-3, and caspase-8 proteins were activated and cleaved in the HCA group, the expression of Bax was upregulated and that of Bcl-2 was downregulated. The expression of apoptosis-related proteins could be reversed by apoptosis inhibition. Compared with the control group, the expression levels of the necroptosis-related proteins RIP1, RIP3, and MLKL were downregulated in the HCA group but were not phosphorylated. The necroptosis inhibitor increased the expression of RIP1 but caused no significant changes in RIP3 and MLKL, and none were phosphorylated.

**Conclusions:**

HCA, as a new regimen of conventional drugs, was a safe and efficacious reinduction salvage strategy in children with refractory AML before HSCT. HCA exhibits the synergistic growth inhibition of AML cells and induces cell death mainly through apoptosis.

## 1. Introduction

The outcomes of children with acute myeloid leukemia (AML) have been significantly improved in recent years because of the application of hematopoietic stem cell transplantation (HSCT), the combination of multiple drugs with chemotherapy, and the rise of novel targeted drugs. However, children with refractory AML still have poor outcomes. The main reason for the formation of refractory AML is the resistance of leukemia cells to chemotherapeutic drugs. The treatment options for refractory leukemia mainly include the use of drugs without cross-resistance, medium and high doses of cytarabine (Ara-C), HSCT, new targeted therapy drugs or biological therapy. Among these treatment regimens, HSCT may be the best choice for refractory patients. It is important to achieve complete remission (CR) before HSCT and longer disease-free survival (DFS) for patients who have no chance of transplantation.

Although there has been significant development of targeted drugs, traditional chemotherapy drugs were mature, stable, and economical. We should not give up the optimization of traditional chemotherapy regimens. Common chemotherapeutic agents for AML include purine analogues, Ara-C, homoharringtonine, and anthracyclines. However, among these chemotherapeutic agents, increasing anthracycline and Ara-C doses may not improve response rates in AML with relapse or nonresponse [1]. Regimens containing homoharringtonine or cladribine with different CR rates of reinduction that range from 0% to 75% have been confirmed to be effective and safe in children with AML [2–8], and they have been reported in a few studies on children with refractory or relapsed AML (RR-AML) [3, 4]. Therefore, it is important to find an optimal combination of traditional chemotherapeutic drugs without increasing the severe damage to the patient's body and achieve effective reduction of minimal residual disease (MRD) and CR.

In this study, we described a new regimen of conventional chemotherapy drugs (homoharringtonine, cladribine, and aclarubicin (HCA)) for refractory AML. The patients were followed up for 23-28 months, and the CR rate and DFS were 100%. Moreover, we examined the effects of HCA on AML cell line proliferation and identified the cell death pathway of these drugs.

## 2. Materials and Methods

### 2.1. Ethics Statement

After the study was approved by the IRB of the Third Xiangya Hospital of Central South University, we retrospectively collected the basic data, clinical indicators, and laboratory indicators of the children treated with the HCA regimen in the Xiangya Hospital or in the Third Xiangya Hospital. All five children enrolled in this study were treated at Xiangya Hospital (Changsha, China).

### 2.2. Treatment

The HCA regimen was as follows: cladribine at 5 mg/m^2^/day as a continuous infusion for 24 hours on days 1–4,aclarubicin at 14 mg/m^2^/day as an intravenous infusion over 2 hours on days 1–4, homoharringtonine at 1 mg/m^2^/day as an intravenous infusion over 3 hours on days 1–7.

Bone marrow (BM) aspirates were collected before each course of HCA and posttreatment on day 28 (±7 days) after HCA to assess the response. Response was assessed by bone marrow aspirates and MRD. Cytogenetic analysis can also be used to assess response status. Discontinue treatment in the event of a serious adverse reaction. Provide supportive care according to local institutional guidelines.

### 2.3. Definitions

CR was defined as less than 5% blasts on BM morphology examination and satisfactory hematological recovery (neutrophil count > 1.0 × 10^9^/L, platelet count > 80 × 10^9^/L). Reaching CR with incomplete hematological recovery was defined as a CRi. Refractory acute myelogenous leukemia was defined as patients who had not achieved CR after at least two courses of previous-line induction chemotherapy [9, 10].

### 2.4. Cell Lines and Cell Cultures

HL60, THP1, and Panc-1 cells were purchased from the Xiangya School of Medicine Type Culture Collection (Changsha, China). RPMI-1640 medium was used to culture HL60 and THP1 cells. DMEM was used to culture Panc-1 cells. All cells were cultured at 37°C with 5% CO_2_ in a cell incubator. Complete medium contained culture medium, 10% FBS and 1% antibiotics.

### 2.5. Antibodies and Reagents

Antibodies against GAPDH (#97166S, 1 : 1000), PARP (#9542S, 1 : 1000), caspase-3 (#9662S, 1 : 1000), cleaved caspase-3 (#9664S, 1 : 1000), caspase-8 (#9746S, 1 : 1000), cleaved caspase-8 (#9496S, 1 : 1000), Bax (#5023S, 1 : 1000), Bcl-2 (#15071S, 1 : 1000), p-RIP1 (Ser166; #65746S, 1 : 1000), RIP3 (#95702S, 1 : 1000), anti-rabbit IgG, HRP-linked antibody (#7074S, 1 : 3000), and anti-mouse IgG, HRP-linked antibody (#7076S, 1 : 3000) were obtained from Cell Signaling Technology (Danvers, MA, USA). Antibodies against MLKL (#ab184718, 1 : 1000), p-MLKL (Ser358; #ab187091, 1 : 1000), RIP1 (#ab72139, 1 : 1000), and p-RIP3 (Ser227; #ab209384, 1 : 1000) were purchased from Abcam (Cambridge, UK). Cladribine, homoharringtonine, Z-VAD-FMK, cycloheximide, chloroquine, ferrostatin-1, and necrostatin-1 were purchased from TargetMol (Shanghai, China). Aclarubicin was obtained from APExBIO (Houston, TX). A stock solution containing cladribine, homoharringtonine, and aclarubicin was prepared in DMSO and kept at -80°C. TNF-alpha was purchased from ABclonal (Wuhan, China).

### 2.6. Growth Inhibition Assay

The inhibition of HL60 or THP1 cell growth was assessed with CCK-8 (Dojindo, Japan). Cells (8 × 10^5^/mL) were seeded in 96-well plates with different concentrations of homoharringtonine, cladribine, and aclarubicin. 10 *μ*L of CCK-8 solution was added to each well after 24 or 48 hours in culture. The 96-well plates were incubated in a cell incubator in the dark for 4-6 hours, and then, the absorbance at 450 nm was measured using a microplate reader (Thermo Multiskan MK3, USA). The cell line experiments were repeated at least three times with three replicates for each trial.

### 2.7. Evaluation of Apoptosis

Cells were treated with different combinations of reagents for 48 hours, collected in EP tubes, washed twice with PBS, and resuspended by adding binding buffer. Apoptotic cells were stained using an Annexin V-FITC/PI Apoptosis Detecton kit (GEnView, Beijing, China). Then, 5 *μ*L of Annexin V-FITC and 5 *μ*L of PI were successively added to the cells according to the instructions. Apoptotic cells were detected using a CytoFLEX flow cytometer (A00-1-1102, Beckman Coulter) after 15-minutes of incubation at room temperature in the dark. Early apoptotic cells showed Annexin V-FITC^+^/PI^−^, and late apoptotic cells showed Annexin V-FITC^+^/PI^+^.

### 2.8. Hoechst/PI Double Staining

HL60 and THP1 cells were treated with different combinations of reagents for 24 hours, Hoechst 33342 was added to the culture medium at a final concentration of 2 *μ*g/mL, incubated at 37°C for 10 minutes, harvested, and washed with PBS. Then, the cells were resuspended in PBS containing PI (10 *μ*g/mL), incubated at 4°C in the dark for 15 minutes, and washed with PBS. The cell death rate was calculated by determining the Hoechst^+^/PI^+^ ratio.

### 2.9. Western Blot Analysis

Cells were collected into EP tubes, washed twice with PBS, lysed using RIPA buffer containing protease and phosphatase inhibitors, incubated on ice for 30 minutes, subjected to high-speed centrifugation and collected from the protein solution. The protein concentration was measured with a BCA (Thermo Fisher Scientific). The calculated protein solution was mixed with 4× SDS sample buffer, boiled at 100 degrees for 10 minutes, and then separated by electrophoresis. After electrophoresis, proteins were transferred to PVDF membranes, blocked with 5% nonfat milk for 1 hour on a shaker, and incubated with primary antibodies overnight at 4°C. Primary antibodies were washed with TBST and then incubated with secondary antibodies for 2 hours at room temperature (RT) on a shaker. Finally, the membranes were washed with TBST, and the proteins were visualized using an ECL kit (GEnView, Beijing, China).The membranes were scanned and analyzed using a gel imager (Bio-Rad-1708195, USA).

## 3. Results

### 3.1. Patient Baseline Characteristics

Between June 2019 and December 2019, a total of 5 AML patients treated with the HCA regimen were reviewed. Patient baseline characteristics are summarized in Table 1. Among the 5 patients, 2 were boys and 3 were girls. The median age at initial diagnosis was 6.3 years (range, 1-13 years). Two patients received the mitoxantrone, Ara-C, and G-CSF (MAG) regimen, and 3 patients received the daunorubicin, Ara-C, and etoposide (DAE) regimen as their first-line induction therapy. These children with AML failed CR after 2-3 cycles of conventional chemotherapy and then received the HCA regimen.

### 3.2. Treatment Administration, Response, and Outcome

Five children with AML who failed to achieve CR after two to three courses of conventional chemotherapy received the HCA regimen for two cycles. The regimen for HCA treatment was as follows: 5 mg/m^2^ cladribine on days 1-4, 1 mg/m^2^ homoharringtonine on days 1-7, and 14 mg/m^2^ aclarubicin on days 1-4.

All patients received the HCA regimen, and the details of the responses and evaluation of hematological can be seen in Table 2. All AML patients achieved an overall response after treatment with the HCA regimen: 4/5 AML patients achieved CR and 1 achieved CRi.

Among the five patients, one child received 2 courses, and one child received 1 course of consolidation chemotherapy without HSCT and was followed up without other treatment. One child received 1 course of consolidation chemotherapy and underwent allogeneic stem cell transplantation (Allo-SCT) 3 months later. The remaining 2 children did not receive consolidation chemotherapy and underwent Allo-SCT at the 6th and 2nd months after achieving CR using the HCA regimen. As of the last follow-up, all 5 patients were in DFS, ranging from 23 to 28 months.

### 3.3. Safety and Toxicity

Five patients received a total of 10 cycles of the HCA regimen. All patients developed severe bone marrow suppression due to their disease and chemotherapy. The median time for neutrophil recovery > 0.5 × 10^9^/L was 15 days, and that for platelet recovery > 20 × 10^9^/L was 6 days. The most common grade 3 to 4 nonhematologic toxicities were febrile neutropenia, pneumonia, sepsis, and intestinal infection. There were treatment-related deaths in this study. The toxicity of the HCA regimen in the children with AML is shown in Table 3.

### 3.4. HCA Synergistically Inhibited HL60 and THP1 Cell Growth

**Figure 1:**
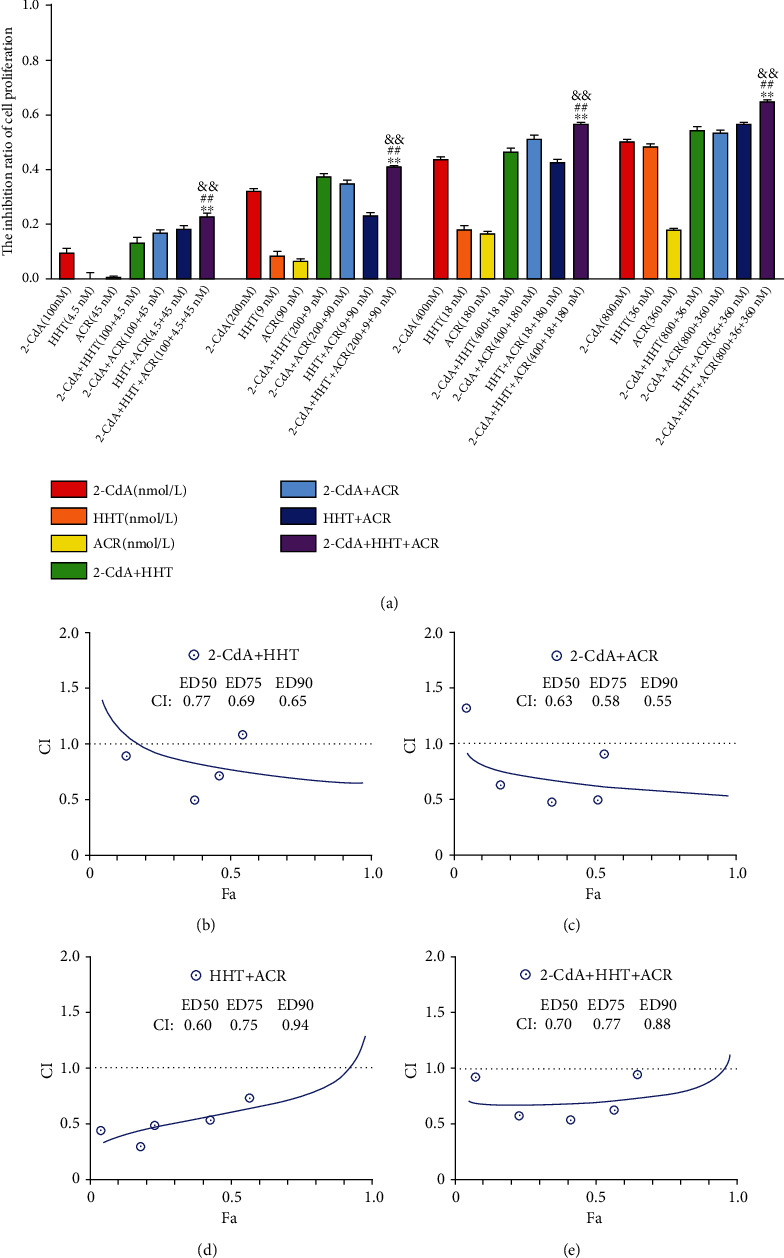
The growth inhibition and CI in HL60 cells treated with different combinations. The rate of growth inhibition induced by cladribine (2-CdA), homoharringtonine (HHT), aclarubicin (ACR), 2-CdA+HHT, 2-CdA+ACR, HHT+ACR, and 2-CdA+HHT+ACR (HCA) in HL60 cells (a) for 24 hours. CI values for 2-CdA+HHT combination treatments at a molar ratio of 1 : 0.045 in HL60 cells (b), 2-CdA+ACR (1 : 0.45) (c), HHT+ACR (10 : 1) (d), and 2-CdA+HHT+ACR (1 : 0.045 : 0.45) (e). ^∗∗^*P* < 0.01, 2-CdA+HHT+ACR vs. 2-CdA+HHT. ^##^*P* < 0.01, 2-CdA+HHT+ACR vs. 2-CdA + ACR. ^&&^*P* < 0.01, 2-CdA+HHT+ACR vs. HHT+ACR.

**Figure 2:**
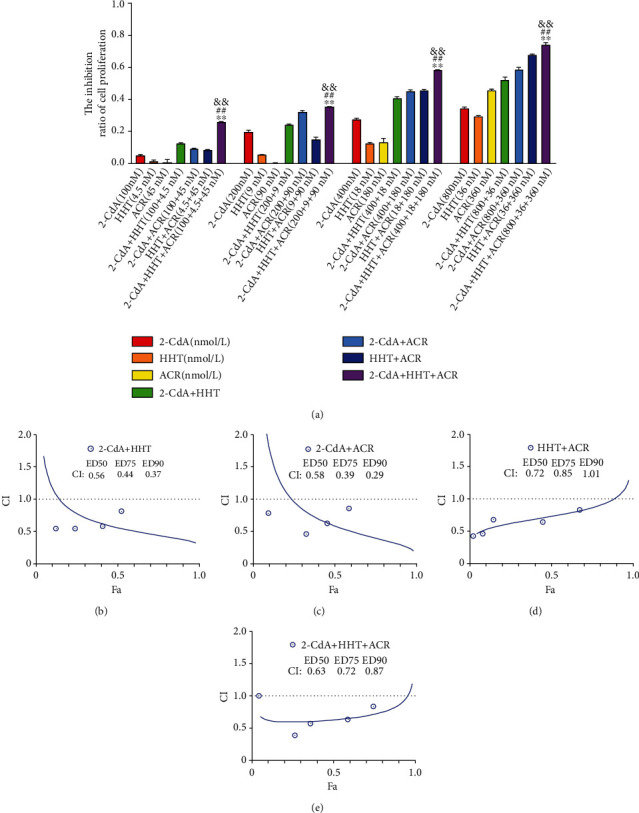
The growth inhibition and CI in THP1 cells treated with different combinations. The rate of growth inhibition induced by 2-CdA, HHT, ACR, 2-CdA+HHT, 2-CdA+ACR, HHT+ACR, and HCA in THP1 cells (a) for 24 hours. CI values for 2-CdA+HHT combination treatments at a molar ratio of 1 : 0.045 in THP1 cells (b), 2-CdA + ACR (1 : 0.45) (c), HHT+ACR (10 : 1) (d), and 2-CdA+HHT+ACR (1 : 0.045 : 0.45) (e). ^∗∗^*P* < 0.01, 2-CdA+HHT+ACR vs. 2-CdA+HHT. ^##^*P* < 0.01, 2-CdA+HHT+ACR vs. 2-CdA+ACR. ^&&^*P* < 0.01, 2-CdA+HHT+ACR vs. HHT+ACR.

Homoharringtonine, cladribine, and aclarubicin are commonly used chemotherapeutic drugs for the treatment of leukemia, and they can inhibit proliferation and induce cell death. To examine whether HCA produces synergistic antitumor activity, HL60 and THP1 cells were treated with various concentrations of the three drugs for 24 or 48 hours. The following concentrations were used: cladribine (100, 200, 400, and 800 nmol/L), homoharringtonine (4.5, 9, 18, and 36 nmol/L), and aclarubicin (45, 90, 180, and 360 nmol/L). The half-maximal inhibitor concentration (IC50) values of cladribine, homoharringtonine, and aclarubicin for HL60 at 24 and 48 hours were 1.18 *μ*mol/L and 0.17 *μ*mol/L, 69.59 nmol/L and 11.8 nmol/L, and 1.69 *μ*mol/L and 0.61 *μ*mol/L, respectively (*n* = 3). The IC50 values of cladribine, homoharringtonine, and aclarubicin for THP1 at 24 and 48 hours were 3.57 *μ*mol/L and 0.22 *μ*mol/L, 110.2 nmol/L and 18.22 nmol/L, and 0.41 *μ*mol/L and 0.33 *μ*mol/L, respectively (*n* = 3). The cytotoxicity of the three drugs to HL60 and THP1 cells was measured with CCK-8 assays. The inhibition of HL60 or THP1 cell growth was concentration dependent. Our data show that HCA has a significantly higher inhibitory effect on AML cells than any monotherapy or two-drug combination (Figures 1(a) and 2(a)) (*P* < 0.01). The combination index (CI) values when HCA was applied to AML cells were analyzed using CompuSyn software, and the results showed a synergistic effect (Figures 1(b)–1(e) and 2(b)–2(e)). Thus, HCA had a synergistic inhibitory effect on the growth of AML cell lines.

### 3.5. HCA Induced Apoptosis in HL60 and THP1 Cells

**Figure 3:**
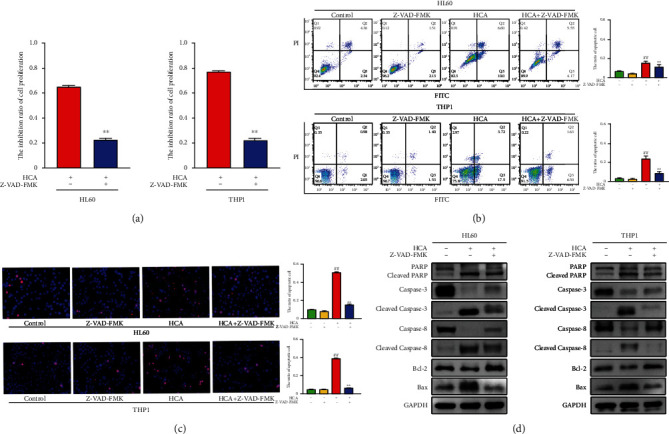
HCA induced apoptosis. HL60 cells and THP1 cells were treated with 2-CdA (800 nM), HHT (36 nM), and ACR (360 nM) for 24 hours. The rate of growth inhibition induced by HCA, with or without 60 *μ*mol/L Z-VAD-FMK in HL60 cells and THP1 cells (a). The rate of HL60 and THP1 apoptotic cells was measured by flow cytometry (b). Hoechst/PI staining showed that HL60 and THP1 cells in the HCA group had the highest number of condensed and/or fragmented nuclei, and it was lower in the Z-VAD-FMK groups (c). Western blot analysis of apoptotic protein expression after 24 hours (d). ^##^*P* < 0.01, HCA vs. control. ^∗∗^*P* < 0.01, HCA+Z-VAD-FMK vs. HCA.

According to the cell inhibition rate, cladribine, homoharringtonine, and aclarubicin had the strongest inhibitory effect on AML cells at concentrations of 800, 36, and 360 nmol/L, respectively, and these concentrations were used in the next experiment. It has been reported that homoharringtonine, cladribine, and aclarubicin all individually induce apoptosis in leukemia cells. In this study, we investigated whether the combination of these three drugs also promotes apoptotic death in AML cells. We found that HCA-induced cell death was suppressed after the addition of the apoptosis inhibitor Z-VAD-FMK (Figure 3(a)). FACS analyses and Hoechst/propidium iodide (PI) staining indicated that HCA could induce apoptosis in AML cells (Figures 3(b) and 3(c)).

Western blotting was used to evaluate the expression of apoptosis-related proteins including Bcl-2, Bax, and caspase family members in AML cells after treatment with HCA for 24 hours. As expected, PARP, caspase-3, and caspase-8 were cleaved in HL60 and THP1 cells after treatment with HCA, indicating that HCA could induce apoptosis in these cell lines (Figure 3(d)). Furthermore, a pronounced increase in Bax and a decrease in Bcl-2 were induced by the combination treatment (Figure 3(d)). The expression patterns of these apoptosis-inducing proteins were reversed after adding Z-VAD-FMK.

**Figure 4:**
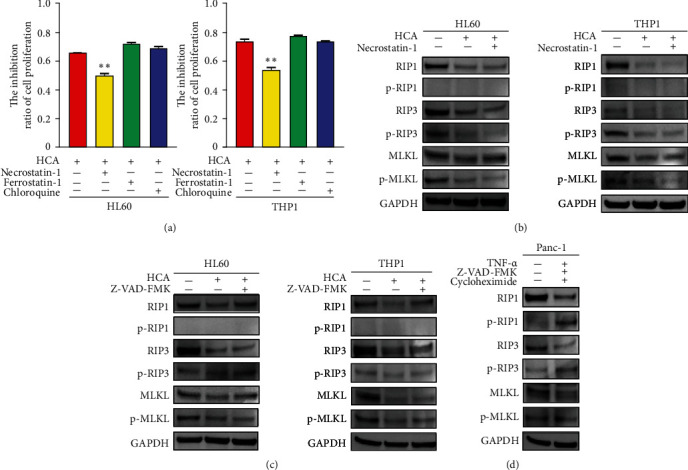
HCA induced other death modes of AML cells. The rate of growth inhibition induced by 2-CdA+HHT+ACR, with or without different inhibitors in HL60 and THP1 cells (a). Western blot analysis of necroptosis protein expression after 24 h (b). Western blot analysis of necroptosis protein expression after 24 hours (c). Western blot analysis of necroptosis protein expression in Panc-1 cells following treatment with or without TNF-alpha (20 ng/mL), cycloheximide (10 ng/mL), and Z-VAD-FMK (20 *μ*M) for 12 hours as positive control (d). ^∗∗^*P* < 0.01, HCA+necrostatin-1 vs. HCA.

To determine whether the combination therapy with HCA also affects other modes of cell death in AML cells, we added necrostatin-1, a necroptosis inhibitor, chloroquine, an autophagy inhibitor, or ferrostatin-1, a ferroptosis inhibitor. As shown, HCA-mediated cell death was partially suppressed only after the addition of necrostatin-1 (Figure 4(a)). Then, Western blotting was used to observe the expression and activation of RIP1, RIP3, and MLKL as biomarkers of necroptosis. Interestingly, the target proteins were not activated but instead were cleaved in HL60 cells and THP1 cells treated with HCA or HCA+necrostatin-1 for 24 hours (Figure 4(b)). To explore whether necroptosis occurs when cell apoptosis is compromised, an apoptosis inhibitor was added when cell death was induced with HCA. We found that the expression level of RIP1 was increased, but there were no changes in the phosphorylation of RIP1, RIP3, and MLKL after the addition of Z-VAD-FMK (Figure 4(c)). Altogether, these results demonstrated that combination therapy with HCA induced apoptotic cell death in AML cells.

## 4. Discussion

Despite enhanced therapeutic management of children with RR-AML, the prognosis remains poor, and their management remains a substantial clinical challenge with few available therapeutic options. It is widely accepted that HSCT is the best treatment option and the only hope to cure them. The mortality rate after HSCT remains high in children who receive repeated intensive chemotherapy. The remission status before transplantation is one of the risk factors associated with the long-term survival rate after transplantation [12–14]. It has been stated that the 5-year overall survival (OS) probabilities of patients undergoing transplantation after second CR, relapse, and primary induction failure are 45%, 20%, and 12%, respectively [15]. The final prognosis of children with refractory AML receiving HSCT without achieving a CR was much worse than that of children with relapsed AML [13]. Intensive salvage second-line therapy can cause increased infection-related mortality in RR-AML, and associated toxicities as well as anticipated future adverse effects must be considered [16]. Investigating optimal reinduction therapies to achieve as many CR as possible and bridge to HSCT thereafter is crucial.

Some reported chemotherapeutic regimens (administered as monotherapies or in combination), such as nucleoside analogs (clofarabine, cladribine, and fludarabine), tyrosine kinase inhibitors (sorafenib), proteasome inhibitors (bortezomib), epigenetic therapies (decitabine), and immunotherapies (gemtuzumab ozogamicin), have been used in children with RR-AML [17]. The fludarabine, Ara-C, and G-CSF (FLAG) combined with anthracycline regimens in children and young adults with RR-AML were reported to produce a CR rate of 69%-74% [18, 19], but the profound myelosuppression, high treatment-related death rate, and final low long-term survival rate have limited the use of this regimen as a standard reinduction chemotherapy protocol for pediatric RR-AML [20, 21].

Cladribine, the first-generation deoxyadenosine analog, has unique antileukemic properties. In the treatment of adult AML, the addition of cladribine to the standard regimen has a better efficacy [22]. For AML patients with relapse or poor risk stratification, the cladribine, Ara-C, and G-CSF (CLAG) regimen could effectively improve CR and prolong OS compared with the FLAG regimen [23]. Cladribine has been found to have activity against AML in children when combined with Ara-C or topotecan [4]. Studies on the CR rate of reinduction therapy with cladribine in pediatric RR-AML are rare, and the results significantly differ. Clinical studies have reported that Ara-C combined with the cladribine regimen did not achieve CR after 2 cycles of treatment in 9 adolescent AML patients [24], while the CLAG regimen achieved a 75% CR rate in 12 children with RR-AML [3]. Although cladribine combined with anthracycline salvage chemotherapy in adult relapsed AML was reported to achieve a relatively high CR rate [25, 26], cladribine plus idarubicin in children with AML experiencing their first relapse achieved a CR rate of only 46% [27].

Aclarubicin is an anthracycline antibiotic isolated from *Streptomyces galilaeus* that exhibits major chemical differences from the conventional anthracyclines daunorubicin (DNR) and adriamycin (ADM). Aclarubicin can not only inhibit the replication and repair of DNA and the synthesis of RNA and proteins [28] but it can also lead to mitochondrial dysfunction [29]. In addition, aclarubicin induces oxidative DNA damage and apoptosis [30]. Aclarubicin-based chemotherapy protocols, Ara-C, aclarubicin, and G-CSF (CAG) regimen, have better efficacy and safety in initially diagnosed or RR-AML [31–33], which is based mainly on observations in adult AML. Data on aclarubicin in pediatric AML are rare. Chen et al. reported that a CAG regimen in children with AML achieved a 38.4% CR rate without severe adverse effects [34].

Homoharringtonine is a plant alkaloid that is widely used to treat adults with initially diagnosed or RR-AML. Homoharringtonine exerts antitumor effects by inhibiting the synthesis of target proteins, such as phospho-eIF4E, SP1/TET1/5hmC, Smad3, and TGF-*β* pathway components, and NF-*κ*B repressing factor [35–38], and promotes apoptosis in leukemia cells. Homoharringtonine-based protocols, such as homoharringtonine, Ara-C, and G-CSF (HAG) and homoharringtonine, Ara-C, and aclarubicin (HAA), appear to be more effective and better tolerated than intensive chemotherapy in the treatment of adult RR-AML [39–43]. Ma et al. reported that HAG+aclarubicin (GHAA), and HAG+pirarubicin (GHTA) are more efficient than conventional HAG without anthracycline, and the efficiency of GHAA/GHTA is positively correlated with B7.1 expression [44]. Satisfactory results can also be obtained when homoharringtonine is used in combination with orafenib or the CAG regimen in the treatment of AML patients [45, 46]. These data reveal the promising potential of HHT as an antileukemic agent. Even children under two years of age with AML can benefit from HHT-containing chemotherapy regimens with tolerable toxicities, such as nausea, vomiting, diarrhea, and mucositis [6, 8]. Few studies have investigated HHT in the treatment of pediatric RR-AML. In a previous study, children with refractory leukemia experienced death after treatment with HHT alone, the reason might be associated with single-drug administration [47].

Identifying new salvage regimens that produce a relatively high CR rate and low toxicity in children with RR-AML is still an area of investigation. For the selection of a treatment regimen for RR-AML, resistance to individual prior therapies and their associated toxicity must be considered. An increased dose of a conventional chemotherapeutic agent, such as Ara-C or daunomycin, is not optimal for pediatric RR-AML because of dose-related toxicities and non-obvious improvements [48]. Based on the above-described results, the efficacy and safety of the CLAG and HAA regimens, and observations for refractory patients at our center who previously received a DAE regimen, an idarubicin, Ara-C, and G-CSF (IAG) regimen or a mitoxantrone, Ara-C, and G-CSF (MAG) regimen as induction chemotherapy, we first administered the HCA regimen, which avoids the use of Ara-C and replaces daunomycin, idarubicin or mitoxantrone with aclarubicin, to children with primary refractory AML. We referred to the CLAG, HAA, and GHAA regimens in adult AML and our previous experiences in the treatment of AML and myelodysplastic syndrome (MDS) and chose 5 mg/m^2^ cladribine on days 1-4 [49], 1 mg/m^2^ homoharringtonine on days 1-7, and 14 mg/m^2^ aclarubicin on days 1-4 as the reinduction protocol for children with primary refractory AML. All five children who did not respond to conventional chemotherapy achieved CR or CRi after two cycles of the HCA regimen, and the CR/CRi rate was 100%. The most common events were severe myelosuppression, febrile neutropenia, and intestinal infection. All of the toxicities were tolerable and acceptable. No neurological or cardiac events or treatment-related deaths occurred during treatment. The median time for platelet recovery > 20 × 10^9^/L was 6 days, which was shorter than that reported with the CLAG regimen (13 days), and the median time for neutrophil recovery > 0.5 × 10^9^/L was 15 days, which was comparable to that reported in a previous study [3]. This suggests that this is a very good combination of traditional drugs.

In vitro, our study showed that homoharringtonine, cladribine, or aclarubicin treatment alone resulted in inhibition of the cell growth of HL60 and THP1 cells in a dose-dependent manner. These findings support the notion that homoharringtonine, cladribine and aclarubicin can inhibit the growth of AML cells [50–53]. To expand our analysis of the synergistic interaction among homoharringtonine, cladribine and aclarubicin, we used HCA to treat the two AML cell lines. Compared with the monotherapies and two-drug combination therapies, HCA exerted a synergistic antiproliferative effect on AML cells (Figures 1 and 2). This study provides persuasive evidence that HCA has a high CR rate in the treatment of refractory AML patients.

Apoptosis is a programmed cell death process and an important mechanism by which chemotherapeutic drugs regulate tumor development [54, 55]. Clinically, chemotherapeutic drugs often achieve therapeutic effects by inducing tumor cell death, such as apoptosis.Cladribine induces apoptosis in human leukemia cells by acting on mitochondria [50], and homoharringtonine regulates leukemia cell differentiation, proliferation, and apoptosis by inhibiting protein synthesis [52, 53]. In our study, HCA significantly induced apoptosis in HL60 and THP1 cells, as confirmed by Annexin V-FITC/PI staining and Western blot analysis (Figure 3).

Necroptosis, a form of cell death defined in recent years, can be inhibited by necrostatin-1. We found that the combined administration of necrostatin-1 or Z-VAD-FMK could reverse HCA-mediated cell death. However, the specific molecular biomarkers evaluated including phosphorylated RIP1, RIP3, and MLKL, which were used to detect the activation of RIP1, RIP3, and MLKL in necroptosis, were not increased after adding Z-VAD-FMK or necrostatin-1 alone to the HCA regimen (Figure 4). This indicated that necroptosis did not occur after HCA administration. In some research, it has been reported that when apoptosis is inhibited, necroptosis is activated as a “fail-safe” mechanism to prevent tumor development [56, 57]. However, in our research, we did not find that necroptosis occurred after apoptosis was inhibited. Furthermore, RIP1, RIP3, and MLKL were found to be degraded after HCA treatment, and RIP1 was able to recover after adding Z-VAD-FMK. The reduced expression of RIP1 and RIP3 confirms the notion that active caspase-8 proteolytically cleaves both RIP1 and RIP3, resulting in their inactivation, which leads to induction of the apoptosis pathway [58]. However, why HCA can affect MLKL expression and why the expression of RIP3 does not recover after the addition of an apoptosis inhibitor requires further investigation. RIP1 dimerization can phosphorylate RIP1, and phosphorylated RIP1 composes complex IIb with FADD and caspase-8 while RIP3 forms complex IIc [59, 60]. Caspase-8 in complex IIb requires activated RIP1 activation, which then mediates RIP1-dependent apoptosis [61, 62]. Complex IIc mediates necroptosis. RIP1-dependent apoptosis may be involved in HCA-induced AML cell death. This may be why necrostatin-1 can inhibit AML cell death.

## 5. Conclusion

In summary, our study suggests that HCA is a very good combination of traditional drugs. HCA synergistically inhibits AML cells and triggers apoptosis. This is the first evaluation of reinduction therapy with the HCA regimen in pediatric primary refractory AML, and the results show that the HCA regimen can be considered a reinduction salvage strategy in children with refractory AML before HSCT or consolidation therapy.

## Figures and Tables

**Table 1:** Patient baseline characteristics.

Characteristics	Patient				
	Case 1	Case 2	Case 3	Case 4	Case 5
Age (yrs)	6.3	1.6	1	8.3	13
Gender	F	M	M	F	F
WBC at diagnosis (×10^9^/L)	2.9	55.5	3.2	0.7	4.4
Hb at diagnosis (g/L)	98	94	106	68	95
Plt at diagnosis (×10^9^/L)	317	178	300	36	29
FAB subtype	AML-M2 with extramedullary infiltration	AML-M2	AML-M5 with extramedullary infiltration	AML-M5	AML-M2
Risk stratification^†^	Intermediate	Favorable	Intermediate	Intermediate	Intermediate
Primary induction chemotherapy	DAE [1], IAE [1]	DAE [2], HA [1]	MAG [2], HA [1]	MAG [2]	DAE [2]
Stage before HCA	Primary induction failure^‡^	Primary induction failure	Primary induction failure	Primary induction failure	Primary induction failure
Bone marrow blast pre-HCA (%)	32.5	16	12.5	20	20.5
Bone marrow blast post-HCA (%)	2.5	2	1	0	2
Karyotype analysis of AML	Normal	Normal	48, XY, +19, +21	Normal	Normal
Translocations/fusion gene analysis	Normal	AML1-ETO+	Normal	Normal	Normal

^†^According to the 2017 risk stratification by the European Leukemia Net (ELN) [11]. ^‡^As defined by lack of response (complete or partial remission) after one course of induction chemotherapy or persistent leukemia after at least two courses of induction chemotherapy. WBC: white blood cell count; Hb: hemoglobin; Plt: platelets; IAE: regimen of idarubicin, etoposide, and Ara-C; HA: regimen of homoharringtonine and Ara-C. The number in “” refers to the number of the treatment cycle.

**Table 2:** Response and outcomes.

Patient	Response	Subsequent therapy (number of cycles)	Outcome	DFS (mon.)
Case 1	CR	HA [1], EA [1]	Alive	28
Case 2	CR	EA [1]	Alive	27
Case 3	CR	HAE [1], Allo-SCT	Alive	25
Case 4	CR	Allo-SCT	Alive	24
Case 5	CRi	Allo-SCT	Alive	23

DFS: disease-free survival; HA: regimen of homoharringtonine and Ara-C; EA: regimen of Ara-C and etoposide; HAE: regimen of homoharringtonine, Ara-C, and etoposide; Allo-SCT: allogeneic stem cell transplantation. The number in “” refers to the number of the treatment cycle.

**Table 3:** Safety and toxicity.

Adverse event	Number of events	%
Hematological toxicities		
Neutropenia (IV)	10	100
Thrombocytopenia (IV)	8	80
Nonhematological toxicities		
Infections		
Febrile neutropenia	6	60
Sepsis	2	20
Pneumonia	2	20
Soft-tissue infection	1	10
Intestinal infection	5	50
Mucositis	2	20
Elevated bilirubin	3	30
Elevated ALT	4	40
Decreased cardiac function	0	0

## Data Availability

The datasets supporting the results of the current study are available from the corresponding author.
